# A Fine-Grained Video Encryption Service Based on the Cloud-Fog-Local Architecture for Public and Private Videos

**DOI:** 10.3390/s19245366

**Published:** 2019-12-05

**Authors:** Hao Li, Zhaoquan Gu, Lianbing Deng, Yi Han, Cheng Yang, Zhihong Tian

**Affiliations:** 1 Post-Doctoral Research Center of Zhuhai Da Hengqin Science and Technology Development Co. Ltd., Zhuhai 519000, China; cuclihao@cuc.edu.cn; 2 Cyberspace Institute of Advanced Technology, Guangzhou University, Guangzhou 510006, China; tianzhihong@gzhu.edu.cn; 3 College of Information Engineering, Communication University of China, Beijing 100024, China; cafeeyang@163.com

**Keywords:** sensor-cloud system, video encryption, public/private video, encryption performance, fine-grained

## Abstract

With the advancement of cloud computing and fog computing, more and more services and data are being moved from local servers to the fog and cloud for processing and storage. Videos are an important part of this movement. However, security issues involved in video moving have drawn wide attention. Although many video-encryption algorithms have been developed to protect local videos, these algorithms fail to solve the new problems faced on the media cloud, such as how to provide a video encryption service to devices with low computing power, how to meet the different encryption requirements for different type of videos, and how to ensure massive video encryption efficiency. To solve these three problems, we propose a cloud-fog-local video encryption framework which consists of a three-layer service model and corresponding key management strategies, a fine-grain video encryption algorithm based on the network abstract layer unit (NALU), and a massive video encryption framework based on Spark. The experiment proves that our proposed solution can meet the different encryption requirements for public videos and private videos. Moreover, in the experiment environment, our encryption algorithm for public videos reaches a speed of 1708 Mbps, and can provide a real-time encryption service for at least 42 channels of 4K-resolution videos.

## 1. Introduction

The sensor cloud system, having gained momentum in the recent years, is identified as an inevitable choice for many problems in the big data age. Multimedia (videos, voices, images, etc.) are major parts of big data, and building a media cloud based on sensors has become a major strategy of the media industry. In a sensor cloud, a large number of sensors are deployed to collect video and audio to the cloud, and finally build a huge multimedia transceiver system. Utilization of the media cloud can alleviate the workload of video service providers and give video users convenient and real-time access to video services.

However, as data are not stored or processed locally, data security emerges as an inevitable concern in deployment and operations of cloud computing platforms [1]. Compared with texts, videos have higher value, not only because they deliver more information, but also because of the copyright and privacy issues involved in videos. Encryption is the most important choice to prevent illegal access to videos and ensure information security [2,3]. Video encryption on media cloud, however, faces many practical problems.

First, how to provide uniform video encryption service for devices with different computing power. Devices connected to the media cloud, such as set-top boxes (STBs), computers, and mobile devices, are diverse and differ in computing power. It is difficult to deploy encryption algorithms that require intensive computing on devices with low computing power. Eiza et al. [4] and Pei et al. [5] proposed video encryption services based on cloud computing. However, as the cloud center is distant from the local server, video encryption on the media cloud usually faces the problem of communication latency [6].

Second, how to meet the different encryption requirements for different type of videos. Generally, there are two types of videos on the media cloud: public videos and private videos. Pubic videos, such as TV programs, user-generated content (UGC) and short videos, aim to attract audience and make profit. This type of video requires encryption but not to the degree of complete unrecognizable. Private videos like surveillance videos, personal videos and conference videos, are highly private, so the content of this type of video should be hidden as much as possible and real-timeliness should be ensured. Whether public video or private video, the implementation of fine-grained encryption for a single video is the basis to meet their different security requirements. Traditional video encryption algorithms in [7,8], however, focus on how to select regions of interest and the encryption efficiency, and fail to pay attention to application in actual scenarios.

Third, how to ensure massive video encryption efficiency. As more 4K- and 8K-resolution videos are produced, the size of videos grow. Assuming that the compression ratio of H.265 videos is between 300:1 and 1000:1 [9], we can obtain the transmission rate of 4K-resolution videos after being encoded by H.265 is about 11~40 Mbps. When the number of videos is large, we have to consider how to increase the encryption efficiency and reduce the latency of video service caused by encryption.

In this paper, we propose a cloud-fog-local video encryption service framework to provide a uniform video encryption service for devices with different computing power. In this framework, the key management is deployed on the cloud layer to produce, store, distribute and destroy keys. The video encryption is deployed on the fog layer and provides encryption service to the nearby terminals of varied computing power. Compared with cloud computing, fog computing is closer to the local users and can provide computing resources of low latency and high elasticity [10]. In order to realize fine-grained video encryption with different security requirement, we propose a fine-grain video encryption algorithm. Based on the network abstract layer unit (NALU), the encryption algorithm provides two security levels, public and private, to achieve fine-grained encryption. In addition, a massive video encryption framework based on Spark ensures the efficiency.

The major contribution of this paper is as follows.

- First, we proposed a three-layer (cloud-fog-local) video encryption framework and designed a corresponding three-layer key management scheme. The framework can provide a uniform video encryption service for devices with different computing power.
- Second, this paper is the first that designs a uniform video encryption algorithm to meet the different encryption requirements for public and private videos, and makes in-depth analysis of the encryption effect. The experiment proves that the algorithm we designed can meet encryption requirements of different types of videos.
- Third, we deployed the video encryption algorithm on Spark, and increased the encryption efficiency and scalability. The experiment proves that the cluster deployed on the two servers under the experiment conditions can realize real-time encryption of at least 42 channels of 4K-resolution videos.

Noted that the focus of our paper is on the mechanism of video encryption, so authentication and authorization are not described in detail. However, when designing the key management scheme, we preset a session key as an interface to connect with secure authentication and authorization strategies (PKI, Kerberos, etc.).

The rest of this paper is arranged as follows. Section 2 introduces the related work on video encryption; Section 3 presents the fine-grained video encryption service model and design details, including the cloud-fog-local framework and its key management scheme, the NALU-based fine-grained video encryption algorithm and the Spark-based massive video encryption framework; Section 4 analyzes the encryption performance of our proposed algorithm; Section 5 is the conclusion of our paper.

## 2. Related Work

Video encryption has long been a focus of the video encoding and information security industries. When the video is large in size, complex in structure and requires real-time transmission, encryption becomes challenging. According to the encrypted content, video encryption can be categorized into full encryption and selective encryption.

**Figure 1:**
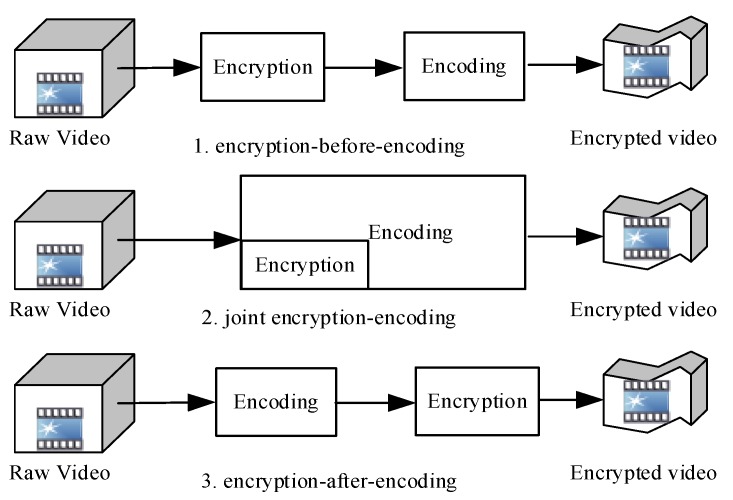
Three selective encryption schemes.

Compared with full encryption, selective encryption has fewer encrypted data and higher encryption efficiency. By the timing of encryption, selective encryption can be categorized into encryption-before-encoding, encryption-after-encoding, and joint encryption-encoding (Figure 1) [3].

In the encryption-before-encoding scheme, people first find the regions of interest and then realize accurate encryption which ensure security of the video and realize format compliance [11,12]. However, the drawback of the encryption-before-encoding scheme is that it seriously reduces the video compression quality. Only when sensitive data in a small range needs to be protected can the encryption-before-encoding scheme have good performance.

In the joint encryption-encoding scheme, there are basically two types. The first type encrypts the original compressed information, such as the intra-frame prediction model, the discrete cosine transform (DCT) coefficients, and motion vectors, as described in [13,14,15,16]. This type of algorithm encrypts videos in the first stage of video encoding, so it can achieve high accuracy in selective encryption and realize complete format compliance, but it will result in severe compression losses. The other type is encryption algorithms based on entropy coding and semantic elements, as described in [17,18,19,20]. This type of algorithm encrypts videos after quantification of the compressed video data, so it achieves lower accuracy in selective encryption than the former type; moreover, when the latter type of algorithm is used, some underlying syntax elements will be inevitably encrypted, and only partial format compliance can be realized. The compression loss will be worse than that incurred by the former type. Because of complex coupling between encoding and encryption, the joint encryption-encoding scheme is more suitable for design of security encoders/decoders.

In the encryption-after-encoding scheme, the advantages are that it can minimize the influence of the video encoding algorithms on encryption and ensure zero compression loss [21,22,23,24]. Its major drawback is that it cannot capture enough information on the video and hence its accuracy in selective encryption is low. The encryption-after-encoding scheme is highly practical because it can be deployed simply after the video encoding system.

Table 1 summarizes the advantages and drawbacks of these three encryption schemes. On the media cloud, Video encryption services prioritize practicability, compression loss and encryption efficiency, but the requirement for encryption accuracy can be lowered. First of all, the media cloud is complex, and video encryption should minimize coupling with the cloud’s own services [25]. Second, when encrypting a massive number of videos, the transmission consumption caused by encryption is unacceptable. Third, encryption efficiency is important because it is correlated to latency of the cloud’s services. Last, low encryption accuracy is acceptable. Encryption accuracy is usually an indicator for performance of selective encryption algorithms (human faces, moving objects, etc.), but privacy is a hard-to-define concept, especially in videos full of all kinds of unstructured information [26]. In practical scenarios, the media cloud only needs to hide privacy information as much as possible. Therefore, encryption accuracy is given a low priority.

## 3. Proposed Fine-Grained Video Encryption Service

Fog computing, shifting intelligence and resources from the remote cloud to edge networks, has the potential of providing low-latency for the communication from sensing data sources to users [27,28]. Abbas et al. proposed a fog security service (FSS) in which the fog layer distributed secret keys to IoT devices [29]. In our section, we proposed a cloud-fog-local video encryption service framework. The framework uses fog computing to provide computing resources to proximate users. To be specific, we completed three parts of work. First, we constructed the cloud-fog-local video service framework and its corresponding key management scheme. Second, we designed a NALU-based fine-grained video encryption algorithm, in which video segments were taken as the encryption content to realize NALU-level fine-grained encryption. Third, we deployed the video encryption algorithm on Spark. These three works will be detailed in the following.

### 3.1. Cloud-Fog-Local Video Encryption Service Architecture

#### 3.1.1. System Architecture

**Figure 2:**
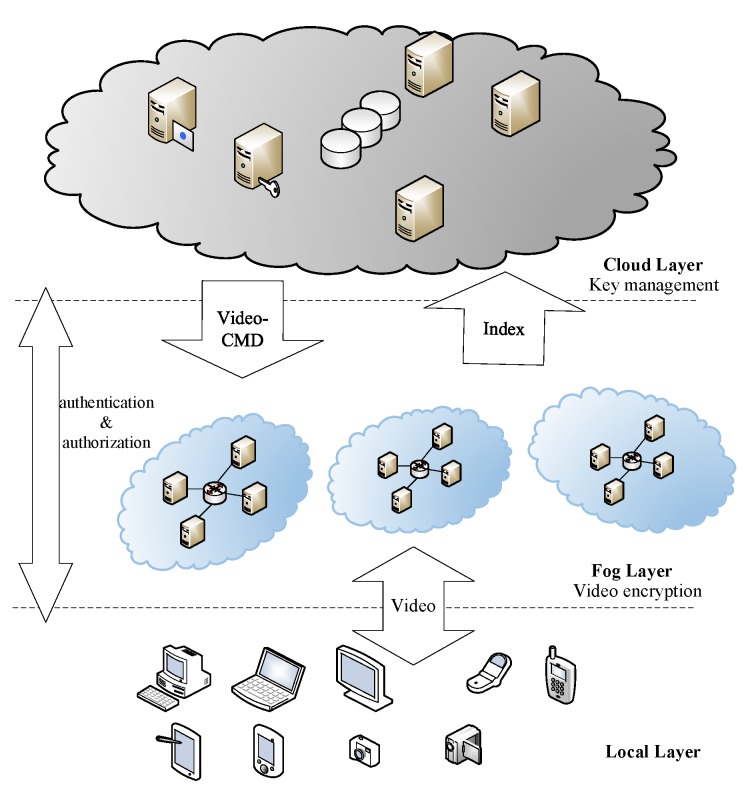
Cloud-Fog-Local Architecture.

In prior studies, we have proposed a “cloud-edge-local” media cloud service hierarchy [30]. Based on [30], we proposed a cloud-fog-local security framework and applied it to video encryption services. Figure 2 shows the architecture of the framework.

**Figure 3:**
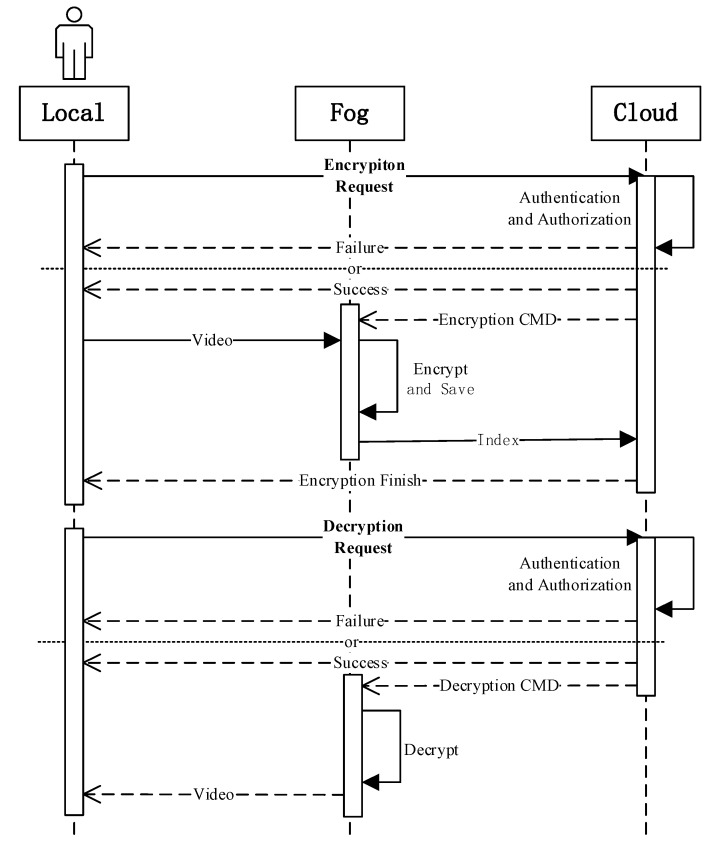
Diagram of encryption and decryption process.

The tasks of each layer in the cloud-fog-local framework are as follows. The cloud layer, as the control center of the whole system, has two tasks. The first is to send video processing commands (file ID, encrypting/decrypting operations, secret keys, etc.) to the fog layer, and recollect the index files similar to m3u8. The other is to verify legitimacy of the devices on the local layer, and complete authentication operations. The fog layer first allocates computing resources for proximate devices, and then encrypts/decrypts the videos. The local layer simulates different user terminals. In the encryption process, it sends video encryption requests to the cloud layer and uploads the videos onto the fog layer. In the decryption process, it sends decryption requests to the cloud layer and receives the decrypted videos from the fog layer. Figure 3 shows the diagram of the encryption and decryption process.

#### 3.1.2. Key Management Scheme

**Figure 4:**
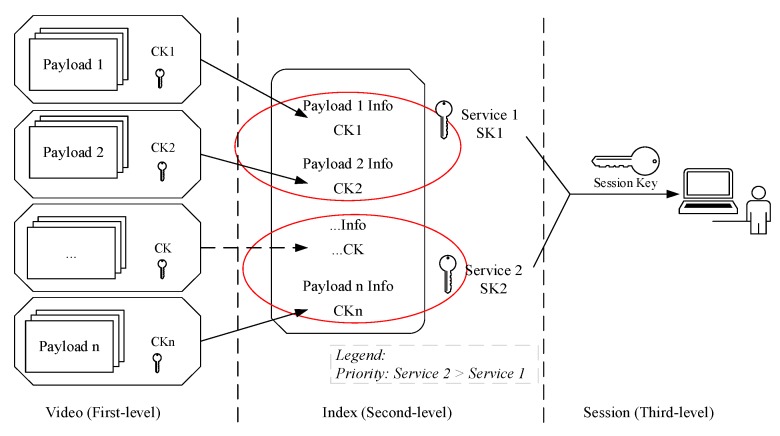
Layered key management architecture.

Encryption of large-scale videos will inevitable produce a large number of secret keys. In order to ensure secure distribution, storage and updating of secret keys, a specific key management scheme is needed [31,32]. We introduced a hierarchical key management scheme corresponding to the three-level architecture of the proposed framework (Figure 4). The scheme has three advantages. First, each NALU is matched with one unique secret key, which is closer to Shannon’s one-time pad. Second, it realizes NAL-level fine-grained video encryption and can satisfy diverse access control. Third, the scheme has three levels of keys: content keys, service keys (also named index keys), and session keys, and is convenient for the management of massive keys.

The first-level keys are content keys (CKs). The payloads in different groups (i.e., different NALUs) are matched with different content keys. The second-level keys are service keys (SKs) that are used to encrypt different parts in the index files. For instance, two services are provided now: Service 1 allows the ordinary users to decrypt only the first six minutes of a video, and Service 2 is provided to VIP users to decrypt the whole video. The index information of the first six minutes of a video will be encrypted by SK1, and the rest index content is encrypted by SK2. When decrypting, ordinary users can only acquire SK1, but VIP users can obtain secret keys for both Service 1 and Service 2 (SK1 and SK2). The third-level keys are session keys that are normally created by security authentication protocols and used to encrypt service keys.

### 3.2. Fine-Grained Encryption Algorithm Based on NALU

Both H.264/AVC and H.265/HEVC use a double-layered structure that consists of a video coding layer (VCL) and a network abstract layer (NAL). The compressed video data on VCL is encapsulated into NALUs (NAL units). NALU consists of two parts: the NALU header and the NALU payload.

The NALU header has a fixed length (one byte for H.264 and two bytes for H.265) and records the content features of NALUs and network information. Thus, to preserve information of NALU headers can guarantee fault tolerance of the video information when transmitted online.

**Figure 5:**
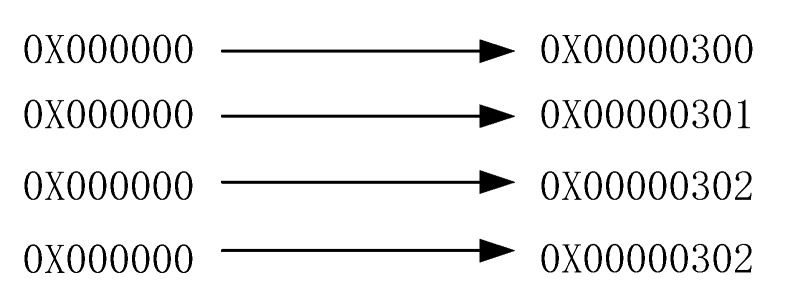
Byte alignment and emulation prevention of raw byte sequence payload (RBSP).

The NALU payload, also termed raw byte sequence payload (RBSP) is full-byte-length information that records video compression data on VCL. In H.264 and H.265, a RBSP is a sequence of bytes (8 bits) filled by a binary string of data bits (SODB) produced by the video encoder. When the last byte of RBSP is filled by SODB, then the *rbsp_trailing_bits* in the format of 100… is added. To avoid emulation between the byte streams in NALU payload and the NALU start code and end code, the RBSP must be byte-aligned and prevent emulation (Figure 5).

The content in RBSP is what we want to encrypt. As the RBSP are divided into different types in the NALU header (*nal-unit-type*), we can analyze the content of different types of RBSP to reduce the content for encryption and increase encryption efficiency. The definition of NALU types differs between H.264 and H.265. In H.264, the video parameters are encapsulated NAL packets termed sequence parameter sets (SPS) and picture parameter sets (PPS); but in H.265, aside from SPS and PPS, the video parameter set (VPS) is introduced to store the global information of the video. Table 2 and Table 3 shows the NALU types in H.264 and H.265, in which NALUs that are considered as IDR (Instantaneous Decoding Refresh) pictures or reference sets have more video information than those not considered as IDR or reference.

For public videos, the encryption algorithm needs to allow format compliance and ensure normal decoding of encrypted videos to stimulate the users’ buying desire, so to encrypt NALUs that contain video content can improve the format compliance and ensure enough cyphertext space. Therefore, for public videos, the units considered as reference pictures are selected for encryption (NALU Type = 5 in H.264; NALU Type = 1, 3, 5, 7, 9, 18, 20, 22~23 in H.265).

For private videos, especially the user’s personal videos, the possibility of video content leakage should be minimized and high encryption efficiency should be ensured. For these videos, NALUs that contain the video’s global information should be selected to encrypt so that the fields to be encrypted can be minimized and no unauthorized decoder can decode the videos. Therefore, for private videos encoded by H.264, the SPS and PPS (NALU type = 8, 9) are selected for encryption; for private videos encoded by H.265, the VPS, SPS and PPS (NALU Type = 32, 33, 34) are selected for encryption, as shown in Table 4.

**Figure 6:**
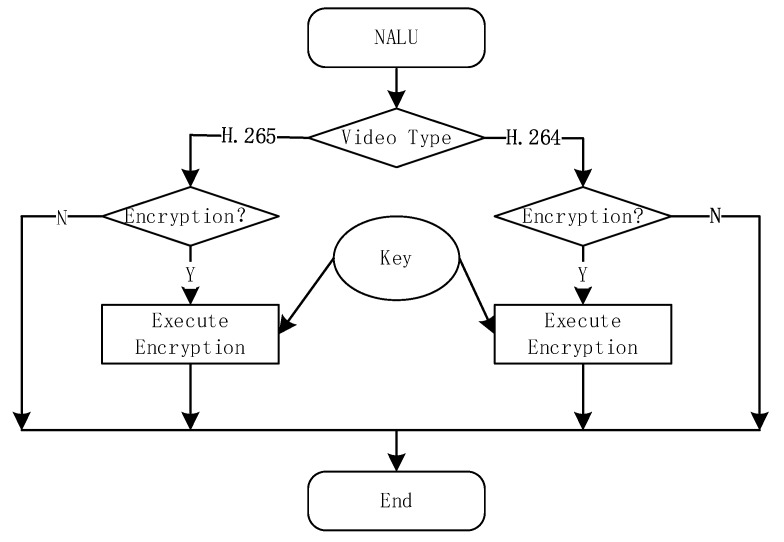
Process of selective encryption.

The last step is the emulation prevention. In order to prevent emulation of encrypted fields with NALU start codes and end codes, the sequence 0 × 000,000 needs to be processed (Figure 5). The AES algorithm is used to encrypt the NALU payload, as shown in Figure 6.

### 3.3. Massive Video Encryption Framework Based on Spark

In this section, Apache Spark is used to execute encryption of massive video. First, massive video NALUs are taken as Spark datasets and form an independent resilient distributed dataset (RDD) for encryption. The video segments are suitable for the high-through-put and fault-tolerant stream processing system of Spark. Second, selective encryption is a complex operation with high computing load, and memory-based processing can increase the encryption efficiency. Last, the Spark framework is scalable and can distribute workload of each node intelligently, hence suitable for the allocate-on-demand encryption service.

Steps to deploy the video selective encryption algorithm onto the Spark framework are as follows:

1. Deploy the Hadoop distributed file system (HDFS) for storing to-be-encrypted videos and encrypted videos;
2. Compile the proposed encryption algorithm as “encryption.so” and deploy it on node servers;
3. Spark executor instances developed by Java call the “encryption.so” from the executor of worker through the JNI interface to execute encryption.

**Figure 7:**
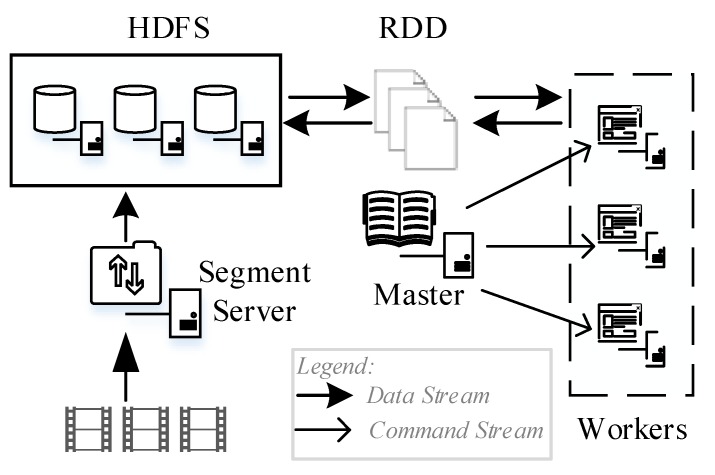
Massive video encryption based on Spark.

The process of massive video encryption based on Spark are as follows (Figure 7):

1. Massive videos are upload and segmented, then the generated, to-be-encrypted segments are stored in HDFS;
2. The Master node receives the tasks and obtains to-be-encrypted video segments from HDFS;
3. The Worker node executes the tasks and encrypt the video segments;
4. The encrypted segments are stored in HDFS, and the encryption is completed.

## 4. Experiments and Analyses

### 4.1. Experiment Environment and Deployment

Table 5 shows the configuration of the Spark cluster servers.

Two servers are used to build the Spark experiment environment, and the gigabit router is used to realize communication between servers. In each server, there are 3 worker nodes, and each node contains 3 CPU cores and 4G of memory.

The realization of the Spark-based massive video selective encryption framework is as follows. The video segments processed by Spark are from HDFS; Spark is in the standalone mode, consisting of Master and Worker. Spark converts the HDFS video segments into resilient distributed datasets (RDDs) required for distributed processing; the *mapToPair* function is used to convert RDDs into *JavaPairRDDs*, and the encryption algorithm is called during the conversion process to encrypt all RDDs. The parameter type of *JavaPairRDD* is *<LongWritable, BytesWritable>*, in which the key of the type *LongWritable* is assigned the video segment ID, the value of the type *BytesWritable* is assigned the video segment; the Master constructs the logic graph of the elastic distributed dataset, that is, the directed acyclic graph (DAG), and transmits the DAG to *DAGScheduler*. The number of partitions in RDD determines the number of tasks, and the Master uses *TaskScheduler*, the shared memory agent node scheduling algorithm, to distribute each element in the distributed dataset to each Worker for processing according to the node performance and use conditions. Encryption of video segments are executed on the Executor on the Worker nodes.

The standard test sequences are used in the experiment, as shown in Table 6.

The YUV-format files are encoded into H.264 or H.265 videos by FFMPG [33], and the video encoding parameters are shown in Table 7. In H.264 encoding process, FFMPEG uses -b:v to adjust the code rate of encoded videos. In H.265 encoding process, FFMPEG uses the quantization parameter (QP) to adjust the code rate. When the QP is 22, 27, 32, and 37, the code rates of H.265 format videos are similar to those presented in Table 8 [20,34].

To ensure credibility of the experiment, we tested the performance of encrypted videos with different code rates and different resolution ratios. Different code rates can be considered as heterogenous network environments with different communication capabilities, and different resolution ratios can simulate terminal devices with different resolution ratios.

A satisfying video encryption algorithm should not only meet requirements for different security levels, but also minimize encryption/decryption-caused computing consumption and reduce encryption-induced loss of compression. Thus, the performance of the proposed fine-grained video encryption framework is discussed with regard to three aspects: security, encryption efficiency and compression loss. Then, we discuss how to realize fine-grained access control of video in our proposed scheme.

### 4.2. Security

In [35], Tang proposed two levels of security for video encryption. The first level is hidden, that is, the encrypted pictures have far poorer quality than the original pictures but are still recognizable. The second level is unrecognizable, that is, the encrypted pictures contain no valid information of the original pictures. Because public videos are to attract potential buyers, encryption of public videos needs to reach the hidden level; however, in the case of private videos, it is hard to define the term of privacy and valid information of the original pictures should be minimized, so encryption of private videos should reach the unrecognizable level. The security of the proposed encryption algorithm is analyzed from two aspects: encryption quality and anti-attack capability.

#### 4.2.1. Encryption Quality

**Figure 8:**
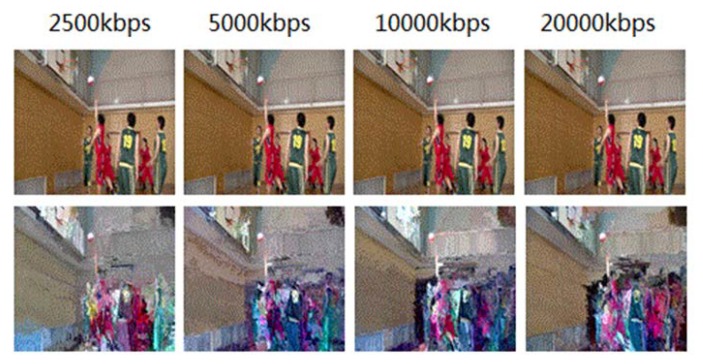
Encryption effect of H.264 video with different code rates (the 351st frame).

**Figure 9:**
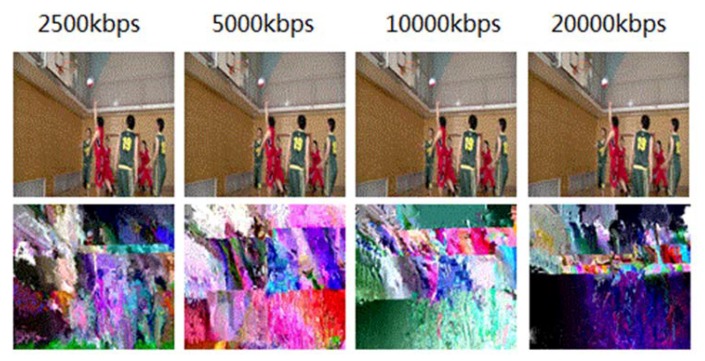
Encryption effect of H.265 video with different code rates (the 351st frame).

Private videos will lose the global information after being encrypted and cannot be played, so it is meaningless to discuss the encryption quality. For public videos, the best effect of encryption is: when an unauthorized user watches an encrypted video, the user can acquire the contour information of the pictures, but have no see to the detailed information. Figure 8 compares the encryption effect of the 351st frame of an H.264 video sequence (BasketballDrive) with different code rates. The original video presents the picture of “shoot”, and the audience can see the “shooting” movements from the encrypted video, but other details including the “shooting player”, “the position of the defender”, and “scoring” are blurred in the encrypted pictures. The visual effect can basically meet the requirement of attracting potential buyers. Figure 9 shows the encryption effect of the 351st frame of an H.265 video sequence (BasketballDrive) with different code rates. Compared with H.264, H.265 video is encrypted with more content, and has more perceptual disruption.

The peak signal-to-noise ratio (PSNR) and the structural similarity index (SSIM) are two major indicators of video encryption quality [36]. PSNR is based on the difference between pixels and sensitive to detailed information; SSIM is concerned with more macroscopic aspects of pictures including luminance, contrast ratio and structure. For public videos that need to attract potential buyers, the encrypted video should have a smaller PSNR, and the SSIM between the encrypted and original videos should be a little larger.

Videos of different resolution ratios are encoded into H.264 and H.265 formats by FFMEPG, and then encrypted by the encryption algorithm we proposed. Tests on a video under different code rates can simulate the encryption effect of video code streams in different network environments, and tests on a video of different resolutions and a fixed code rate (5000 kbps) can simulate the encryption effect of video streams on devices with different resolution ratios.

**Figure 10:**
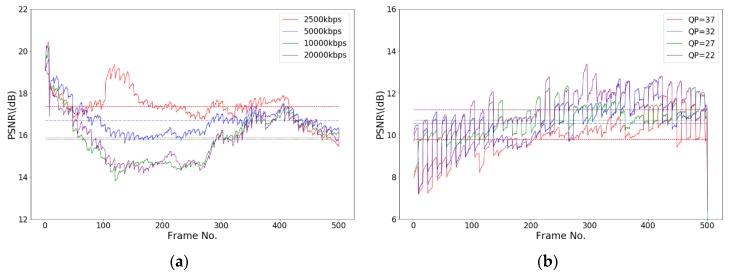
Peak signal-to-noise ratio (PSNR) of encrypted videos with different code rates. (**a**) PSNR of encrypted H.264 format videos with different rates; (**b**) PSNR of encrypted H.265 format videos with different rates.

Typical PSNR values for good quality pictures range between 30 and 40 dB [37]. Figure 10a shows that the PSNR values of an encrypted H.264 video (BasketballDrive) with different code rates are between 14 to 21 dB. Figure 10b shows that the PSNR values of an encrypted H.265 video (BasketballDrive) with different code rates are between 7 and 14 dB. By comparing these two figures, we reach two conclusions: first, encrypted pictures show notable losses in the value of PSNR compared with the original pictures; second, encrypted H.265 videos have a smaller PSNR value than encrypted H.264 videos.

**Figure 11:**
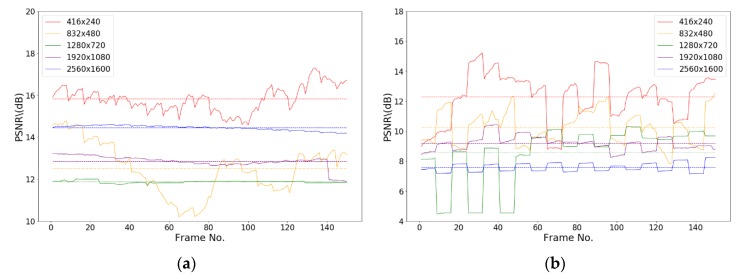
PSNR of encrypted videos of different resolution ratios. (**a**) PSNR of encrypted H.264 format videos with different resolutions; (**b**) PSNR of encrypted H.265 format videos with different resolutions.

Figure 11 shows the test results on videos with different resolution ratios. Videos of five different resolution ratios, 2560 × 1600, 1920 × 1080, 1280 × 720, 832 × 480, and 416 × 240, are tested. Figure 11a shows that the PSNR values of an encrypted H.264 video with different resolution ratios are between 10 and 18 dB, and the encrypted pictures suffer considerable distortion. We encoded the test sequence into H.265 videos and encrypted the videos according to the security standard for public videos. The obtained PSNR values, as shown in Figure 11b, are between 4 and 15 dB. The encryption effect is better than that of H.264 videos, and the distortion of pictures is severe.

SSIM is a metric to assess similarity of two images and has been widely used in video quality assessment. In [38], Yeung et al. argue that SSIM is a more accurate metric than PSNR for image quality assessment. The value of SSIM is between 0 and 1. The closer the SSIM value approaches 0, the less similarity there is between the original image and the encrypted image. As Table 9 shows, the average SSIM value of the BasketballDrive.H.264 video with different code rates before and after encryption is 0.64. Table 10 shows the SSIM values of encrypted images and original images when a video of different resolution ratios is encrypted. Regardless of the type of compression, encryption will inevitably lead to reduction in the SSIM value. The SSIM value of H.265 videos are smaller than that of H.264 videos.

As the experiment result shows, videos encrypted by our encryption algorithm according to the security standard for public videos have small average values of PSNR (16.43 dB for H.264 videos and 10.57 dB for H.265 videos) and high average values of SSIM (0.61 for H.264 videos and 0.32 for H.265 videos). Table 11 compares the encryption effect of our algorithm with that of other algorithms proposed in recent studies. For an H.264 video, our algorithm achieves a PSNR value lower than that obtained in [39] and similar to those in [37,40], but a higher SSIM value. For an H.265 video, our algorithm achieves a PSNR value lower than those in [41,42], but a higher or similar SSIM value. This indicates that when our algorithm is used to encrypt public videos, unauthorized users can get more contour information and less detailed information, so it can meet the requirement of attracting potential buyers.

#### 4.2.2. Brute-force Attacks

To resist brute-force attacks, video encryption should have large key space and ciphertext space. Our proposed massive video selective encryption algorithm uses AES to encrypt video segments, the key length is 128 bits, and the key space reaches 2^128^, which is large enough.

There are two encryption levels: one is the private-video encryption level and the other is public-video encryption level. As the requirements for encryption efficiency differ among videos, two encryption levels have different ciphertext space.

**Private-video encryption level**. Sequence parameter sets and picture parameters sets (video parameter sets are included in H.265 videos) are encrypted in the private video encryption scheme. Parameters with a GOP size of 8 are chosen for encoding. The frame per second (fps) is set as 24. If HLS segments a video into segments of 10 s [43], the frames included in each video slice is 10×24=240. In H.264 videos, the length of the sequence parameter set (SPS) is at least 31 bits (calculated by the known fixed length, same for the following), and the length of PPS is at least 9 bits. In H.265 videos, the length of VPS is at least 106 bits, SPS is at least 40 bits, and PPS is at least 27 bits, which are notably longer than the length of encrypted content in H.264 videos. The size of ciphertext space with video slices as the encryption unit is at least $2^{30\times \left(31+9\right)}=2^{1200}$, and the ciphertext space for private-video encryption is large enough. Table 12 shows that the encrypted content of videos with different resolutions at the private-video encryption level.

**Public-video encryption level**. The encryption space ratio (ESR) is calculated as the ratio of the encrypted bits to all the bits of a compressed video bitstream. Table 13 shows the ESR of different resolution videos when the video bit rate is 5000 kbps. As the resolution increases, the ESR value also increases. It is shown that the more details the video contains, the stronger the ability of public-video encryption to resist brute-force attacks.

In summary, our proposed algorithm has large key space and ciphertext space, so it can defend against brute-force attacks and replacement attacks.

#### 4.2.3. Differential Attack

**Figure 12:**
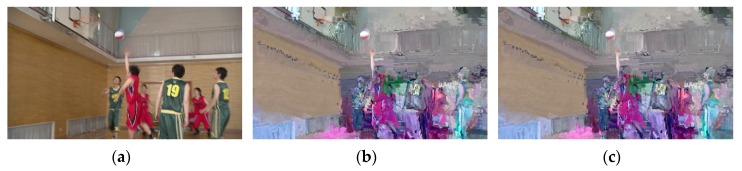
Key sensitivity analysis. (**a**) Original frame; (**b**) Encrypted with K1; (**c**) Decrypted by K2.

In differential attack, the attackers try to guess the keys by investigation of the encrypted bitstream streams. Video encryption algorithm should be sensitive to keys. A slight change of decryption key will make the ciphertext completely unable to decrypt. In our proposed encryption scheme, when the decryption key K2 is only 1 bit different from the encryption K1, the video cannot be decrypted correctly. Figure 12 shows the decrypted image of the 351st frame of the BasketballDrive encoded by H.264. With the original frame as a reference, the PSNR value of the encrypted frame is 13.371646 and the PSNR value of the corresponding incorrect decrypted frame is 13.313982. Using the wrong key to decrypt the video cannot only restore the video, but also make the quality of the video worse.

#### 4.2.4. Known-Plaintext Attack

In this type of attacks, the attackers use the known plaintext and the corresponding ciphertext to obtain the key or the whole plaintext. However, our proposed algorithm has enough ciphertext space. For example, the test sequence “BasketballDrive” contains 501 frames. The compressed video encoded by H.264 according to Table 7 will have at least 63 IDR frames, and a total of 3,060,512 bytes data will be encrypted. Such a large amount of data makes it difficult for attackers to guess the whole plaintext.

#### 4.2.5. Interference Attack

**Figure 13:**
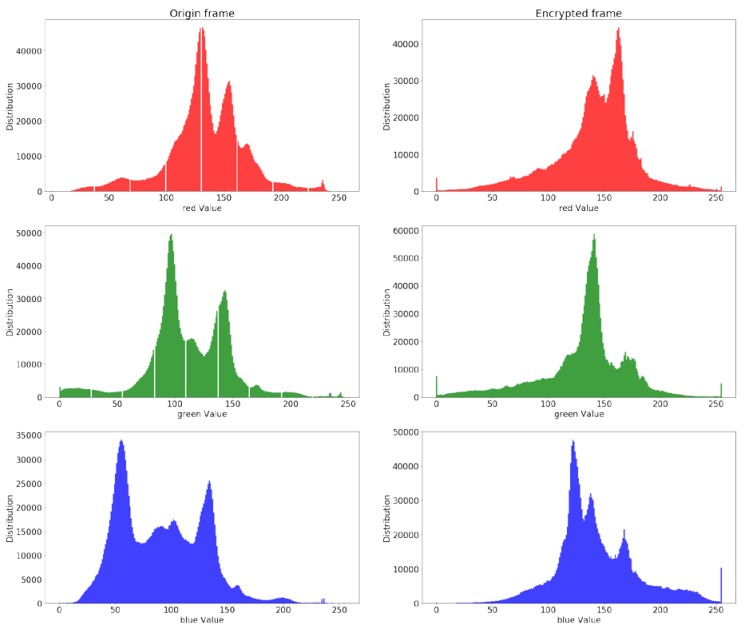
Distribution comparison between the original 351st frame and the corresponding encrypted frame.

Selectively encrypted video is vulnerable to interference attack [40]. Attackers can infer the presence of an object in any one of the R, G, B domains. Figure 13 shows that the pixel distribution histogram of R, G, B channels before and after the 351st frame is encrypted. In the three channels, the pixel distribution of the encrypted frame is distorted to varying degrees. It illustrates that the proposed algorithm can resist the interference attack.

### 4.3. Encryption Efficiency

Massive video encryption is provided to video manufacturers and individuals that are sensitive to encryption efficiency, and scenarios like video conferences and live sports have high standard for real-time transmission. Moreover, as the cloud computing service is very flexible, the encryption service should have high flexibility and scalability [44].

**Figure 14:**
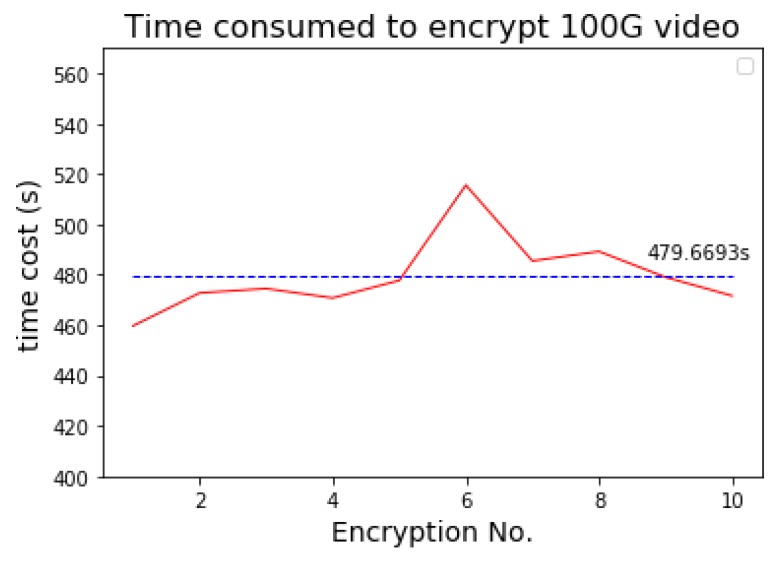
Encryption efficiency.

We use the Spark streaming processing system to extend the selective video encryption algorithm. In the experiment, we segment the 100G video with 120 s as the fixed unit, and execute the encryption algorithm for ten times. Figure 14 shows the time consumed by each encryption operation. In the experiment environment, the public-video encryption algorithm spends an average of 479.6693 s to complete encryption of the 100G video content, so the average encryption speed is 1708 Mbps. The code rate of a 4K-resolution video compressed by H.265 is 11~40 Mbps. Therefore, in the experiment environment, the Spark cluster can meet the encryption requirement for 42 channels of 4K-resolution videos. Meanwhile, as the size of the Spark cluster grows and the resource scheduling and optimizing algorithm improves, the encryption efficiency will improve.

### 4.4. Loss of Compression

For massive videos, loss of compression will cause large consumption of the storage space and transmission bandwidth. The massive video selective encryption algorithm we propose executes encryption after video encoding, which can in theory reduce the loss of compression to 0. By comparing the size of videos before and after encryption (Table 14), we consider that the massive video selective encryption algorithm we propose causes no loss of compression.

### 4.5. Fine-Grained Access Control

Massive video fine-grained access control allows operators to develop more flexible services, but increases the workload for key management. The service framework we propose uses a layered key management scheme. In this scheme, the video encryption layer takes the NALU as the basic encryption unit, each NALU is matched with one content key, and it realizes NALU-level fine-grained encryption and stores video information and corresponding content keys into the video index. In the service layer, the different parts in the encrypted video’s index file are encrypted by the service keys which are generated according to different services. In the authentication & authorization layer, the session keys are built between users and the server, and then used to transport service keys to the corresponding users. In this way, fine-grained access to a particular part of one video is realized. Because massive amounts of content keys are stored in the index, the key management module only needs to manage a few service keys.

**Figure 15:**
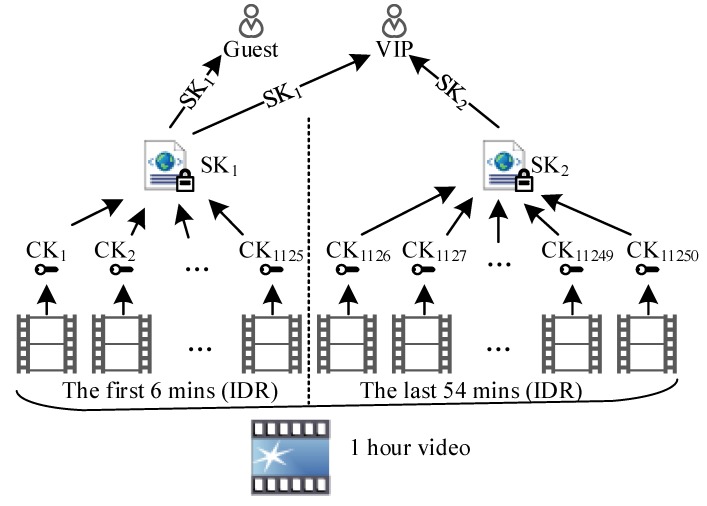
A fine-grained access control scenario.

Figure 15 describes a common fine-grained access control scenario. In this scenario, there is a 1-h video. The first six minutes of the video can be freely viewed by tourists, and the rest can only be viewed by VIP users. Suppose the video play speed is 25 frames per second and the raw video contains 90,000 frames. When the GOP is 8, the whole video has at least 11,250 IDR frames, corresponding to 11,250 CKs. The described scenario has two access levels: (1) the first 6 min; (2) the last 56 min. The 1125 CKs corresponding to the first 6 min video will be encrypted by a service key SK1, and the rest will be encrypted by other service key SK2. The tourists can only get SK1, so they can only play the first 6 min of video. VIP users can get both of the two SKs, so they can watch the whole video. Unlike DES, AES algorithm does not have weak keys. A large number of CKs are generated by the video encryption algorithm itself through a random function. The CKs are saved as index files which will be encrypted by SKs. The key management center only needs to generate and manage two keys, SK1 and SK2. When the access control policy is changed, only two keys needs to be updated or revoked. The burden of the key management center is greatly reduced. Because the encryption content of each CK is very few and only one NALU, the proposed scheme can support more fine-grained and complex access control policy easily.

## 5. Conclusions and Future Work

Video content contains more information than textual content, but faces more problems with regard to copyright and privacy leakage, so it is important to ensure security when the videos are transmitted to the cloud server. Terminal devices, due to limited computing capability, are not suitable for large-scale encryption, and the services have different requirements for real-timeliness and encryption security. To solve these problems, we propose a fine-grained video encryption service that consists of the cloud-fog-local framework, a fine-grained video encryption algorithm based on NALU and a massive video encryption framework based on Spark. Compared with other cloud computing services, our proposed service deploys video encryption on the fog layer that is closer to the local servers, and the cloud layer is only responsible for key management and authority control, so the framework reduces latency of video services. The experiment and analysis verify that our proposed video encryption algorithm can meet the security requirements for both public videos and private videos, and incurs no loss of compression. The encryption speed of encrypting public videos by using the Spark cluster in the experiment environment reaches 1708 Mbps, so it can meet real-time encryption for at least 42 channels of 4K-resolution videos.

In future, our main work will include the following two aspects. First, for the private-video encryption level, we have paid more attention to the efficiency than the security. However, it is necessary to further explore its security capability. Based on the security analysis, we will improve the private video encryption schemes. Second, although the layered key management mechanism can greatly save the workload of the key management center, there is also a new problem, namely that the video encryption key CK cannot be directly updated and revoked. Part of our future work will be focused on how to realize the periodic, automatic and efficient update of CKs.

## Figures and Tables

**Table 1:** Comparison of different encryption schemes.

Performance	Encryption-before-Encoding	Joint-Encryption-Encoding	Encryption-after-Encoding
Encryption accuracy	high	medium	low
Encryption efficiency	low	medium	high
Compression loss	high	medium	low
Practicability	medium	low	high

**Table 2:** Network abstract layer unit (NALU) types in H.264.

NAL Type	NAL Type Description	NAL Type	NAL Type Description
0	Unused	8	PPS
1	Non-IDR picture, no partitioning	9	Delimiter
2	Non-IDR picture, Slice Partition A	10	Sequence end
3	Non-IDR picture, Slice Partition B	11	Stream end
4	Non-IDR picture, Slice Partition C	12	Fill
5	IDR picture slice	13~23	Preserve
6	Supplementary enhancement information unit (SEI)	24~31	Unused
7	SPS		

**Table 3:** NALU types in H.265.

NAL Type	Reference or Not	NAL Type	Reference or Not	NAL Type	Reference or Not
0	N	8	N	20	Y(IDR)
1	Y	9	Y	21	Possible
2	N	10~14	N	22~23	Y
3	Y	15	Possible	24~31	N
4	N	16	Possible	32	VPS
5	Y	17	Possible	33	SPS
6	N	18	Y	34	PPS
7	Y	19	Possible		

**Table 4:** Encryption schemes of different security levels.

Video Attributes	H.264 Encryption Content (NALU Type)	H.265 Encryption Content (NALU Type)
Public video	5	1, 3, 5, 7, 9, 18, 20, 22~23
Private video	8,9	32, 33, 34

**Table 5:** Server configuration.

Item	Configuration (for Each Server)
CPU	24 cores
Memory	32 GB
DISC	100 GB
Operating System	CentOS 7.0
Hadoop	Hadoop2.7.1
Spark	Spark1.6
Java	JDK1.7.051
Number of nodes	3
CPU cores for each node	4
Memory for each node	4 G

**Table 6:** List of test sequences.

Video Name	Frame No.	Frame Rate	Resolution	Format
Traffic	150	30 fps	2560 × 1260	YUV
BasketballDrive	500	50 fps	1920 × 1080	YUV
Kimono1	240	24 fps	1920 × 1080	YUV
Cactus	500	50 fps	1920 × 1080	YUV
KristenAndSara	600	60 fps	1280 × 720	YUV
Johnny	600	60 fps	1280 × 720	YUV
Flowervase	300	30 fps	832 × 480	YUV
PartyScene	500	50 fps	832 × 480	YUV
Keiba	300	30 fps	416 × 240	YUV
RaceHorses	300	30 fps	416 × 240	YUV

**Table 7:** Video encoding parameters.

Video Attribute	H. 264 Parameters	Value	H.265 Parameters	Value
Encoding method	-vcodec	h264	-c:v	libh265
Code rate control	-b:v	Table 8	-x265-params qp	22, 27, 32, 37
Group of pictures (GOP)	-g	8	-x265-params keyint	8
Use of B-frames	-bf	2		libh265

**Table 8:** Different code rates for encoding the same video.

Sequence	Rate 1(QP = 22)	Rate 2(QP = 27)	Rate 3(QP = 32)	Rate 4(QP = 37)
2560 × 1260	30000 kbps	20000 kbps	10000 kbps	5000 kbps
1920 × 1080	20000 kbps	10000 kbps	5000 kbps	2500 kbps
1280 × 720	10000 kbps	5000 kbps	2500 kbps	1000 kbps
832 × 480	10000 kbps	5000 kbps	2500 kbps	1000 kbps
416 × 240	5000 kbps	2500 kbps	1000 kbps	500 kbps

**Table 9:** Structural similarity index (SSIM, average) of encrypted videos with different code rates.

Sequence	2500kpbs/QP = 37	5000kpbs/QP = 32	10000kpbs/QP = 27	20000kpbs/QP = 22
H.264	0.692	0.646	0.616	0.611
H.265	0.275	0.347	0.386	0.415

**Table 10:** SSIM ALL (average) of encrypted videos of different resolution ratios.

Sequence	416 × 240	832 × 480	1280 × 720	1920 × 1080
H.264	0.536	0.600	0.579	0.607
H.265	0.407	0.406	0.320	0.248

**Table 11:** Comparison of our proposed algorithm with other algorithms.

Sequence		Proposed	[37]	[39]	[40]	[41]	[42]
H.264	PSNR	16.43	~14	**~20**	15.22	--	--
	SSIM	0.61	**~0.2**	--	**0.183**	--	--
H.265	PSNR	10.57	--	--	--	**10.79**	**11.06**
	SSIM	0.35	--	--	--	**0.11**	**0.34**

**Table 12:** Encryption depth in Private-video encryption.

Sequence	H.264	H.265
Keiba	1026 bytes	2574 bytes
Flowervase	1026 bytes	2652 bytes
KristenAndSara	899 bytes	5025 bytes
Kimono1	2100 bytes	2139 bytes
Traffic	532 bytes	1311 bytes

**Table 13:** Encryption space ratio (ESR) in public-video encryption level.

Sequence	H.264	H.265
Keiba	23.70%	35.02%
Flowervase	40.48%	43.87%
KristenAndSara	52.80%	58.28%
Kimono1	55.80%	46.66%
Traffic	79.43%	76.48%

**Table 14:** Video size before and after encryption.

Test Sequence	Encoding Format	Encryption Level	Pre-Encryption Size (Byte)	Post-Encryption Size (Byte)
Johnny.yuv	H.264	Public video	15,686,244	15,686,244
ParkScene.yuv	H.264	Private video	12,743,282	12,743,282
Cactus.yuv	H.265	Public video	11,564,461	11,564,461
RaceHorses.yuv	H.265	Private video	3,034,798	3,034,798
